# The Inhibitory Concentration of Natural Food Preservatives May Be Biased by the Determination Methods

**DOI:** 10.3390/foods10051009

**Published:** 2021-05-06

**Authors:** Joana Gomes, Joana Barbosa, Paula Teixeira

**Affiliations:** Escola Superior de Biotecnologia, CBQF-Centro de Biotecnologia e Química Fina–Laboratório Associado, Universidade Católica Portuguesa, Rua Diogo Botelho 1327, 4169-005 Porto, Portugal; joanamcg@hotmail.com (J.G.); pcteixeira@porto.ucp.pt (P.T.)

**Keywords:** agar dilution, antimicrobial activity, chitosan, drop diffusion on agar, food preservatives, propolis, nisin

## Abstract

The demand for natural antimicrobials as food preservatives has increased due to the growing interest of the population for a healthy lifestyle. The application of screening methods to identify the antimicrobial activity of natural compounds is of great importance. The in vitro determination of antimicrobial activity requires determining their minimum inhibitory concentrations to assess microbial susceptibility. This study aimed to evaluate the minimum inhibitory concentrations of three natural antimicrobial compounds—chitosan, ethanolic propolis extract, and nisin—against 37 microorganisms (different pathogens and spoilage microorganisms) by the methods of agar dilution and drop diffusion on agar. Culture media at different pH values were used for both methods to simulate different food products. Most of the microorganisms were inhibited by chitosan (0.5% *w/v*) and propolis (10 mg/mL), and most of the Gram-positive bacteria by nisin (25 μg/mL). Different pH values and the in vitro method used influenced the inhibition of each compound. Generally, lower minimum inhibitory concentrations were observed at lower pH values and for the agar dilution method. Furthermore, some microorganisms inhibited by the compounds on the agar dilution method were not inhibited by the same compounds and at the same concentrations on the drop diffusion technique. This study reinforces the need for using defined standard methods for the in vitro determination of minimum inhibitory concentrations. Natural compounds with potential antimicrobial action are a bet on food preservation. The use of standard techniques such as those used for antimicrobials of clinical applications are crucial to compare results obtained in different studies and different matrices.

## 1. Introduction

With the increasing concern of consumers for a healthier lifestyle, natural compounds have been a prominent choice. Several natural compounds are attractive as food preservatives due to their biocompatibility, biodegradability, safety, low toxicity, and cost efficiency [1]. They are also attractive due to their antimicrobial properties against a broad range of pathogenic microorganisms [2,3]. Many natural compounds also have exciting features to develop greener packaging to reduce pollution caused by nonbiodegradable materials [4]. In this sense, these compounds can be used to prevent microbial food spoilage and/or inhibit the growth of pathogenic microorganisms [5,6]. 

The interest in natural antimicrobials has grown because of different factors, including the misuse of antibiotics, which increased the number of resistant microorganisms and consumer awareness about the safety of chemically synthesized additives [7]. Natural compounds may be an alternative to chemical preservatives, not altering the sensory and nutritional attributes of foods and playing an essential role in food safety [8]. Natural antimicrobials from bacteria, animals, plants, and resins have been considered as potential replacements for chemical preservatives [9]. Some examples are chitosan, propolis, and nisin [9,10,11,12]. These compounds can be directly added into the product formulation, coated on the surface, or incorporated into the packaging material [13]. Chitosan is a polymer with antibacterial, nontoxic, biodegradable, and biocompatible activity [10]. It is obtained by deacetylation of chitin [14], a structural polysaccharide derived from the exoskeleton of crustaceans, insects, and the cell wall of fungi [9]. This compound can inhibit the growth of fungi and bacteria [15] and can be used in food, biomedical, and agricultural industries [16]. Propolis is a natural product composed of various amounts of resins and beeswax collected by honeybees [9]. The ethanolic propolis extract is a product rich in phenolic acids and flavonoids that confer different biological activities, such as antimicrobial, antioxidant, and antitumoral activities [9,11]. Nisin is a bacteriocin produced by Gram-positive bacteria belonging to the lactic acid bacteria group [12]. It acts through the formation of pores in the cytoplasmic membrane of the susceptible cells, causing the dissipation of the membrane potential [17]. Nisin has a broad-spectrum activity against different Gram-positive bacteria [9].

Several authors have been studying the antimicrobial activity of natural compounds against pure cultures of pathogens [2,18,19,20] using different methods, such as disk diffusion, drop diffusion, well diffusion, and dilution in agar or liquid medium, some of which are standardized by the Clinical and Laboratory Standards Institute (CLSI) or by the European Committee on Antimicrobial Susceptibility Testing (EUCAST). The agar dilution method involves incorporating various desired concentrations of the antimicrobial agents in an agar medium, followed by inoculation of the microbial inoculum on the surface of the agar plate [21]. The agar diffusion technique is used to determine the minimum inhibitory concentration on solid media [22], in which diffusion in agar refers to the movement of the molecules through the matrix formed by agar gelling [23]. The agar drop diffusion method (also called the drop method) involves inoculation of the inoculum on the surface of the agar plate, followed by the addition of several drops of the antimicrobial solutions at the desired concentrations, diffusing into the agar medium with the inoculum [2]. The interpretation of the results obtained in the agar diffusion method is based on the assumption that the compound diffuses freely in the solid compound, but, in many cases, this may not happen, giving rise to errors [22]. The lack of a standardized method for the in vitro testing of antimicrobial activity of natural compounds is very limiting due to the impossibility of comparing results from different samples or even from various studies. 

The objective of this work was to compare the minimum inhibitory concentrations of three natural antimicrobials (ethanolic extract of propolis, chitosan, and nisin) at different pH values against 37 microorganisms (21 Gram-positive bacteria, 15 Gram-negative bacteria, and one yeast) using two different assays: agar dilution and drop diffusion on agar. With this study, it will be possible to understand the importance of using defined in vitro techniques to determine the minimum inhibitory concentrations of natural compounds, analyzing whether different methods and conditions provide different results. Standardizing those in vitro techniques will be crucial for studying new antimicrobial compounds before their application in different food matrices.

## 2. Materials and Methods

### 2.1. Microbial Cultures and Growth Conditions

Isolates used in this study (Table 1) were selected in order to include several foodborne pathogens and spoilage microorganisms commonly found in different foods. All isolates, except *Clostridium* spp., were grown in Trypto-Casein Soy Agar with Yeast Extract (0.6% *w/v*) (TSA-YE; Biokar diagnostics, Beauvais, France) at 37 °C under aerobic conditions, for 24 h and stored at −80 °C in Trypto-Casein Soy Broth (TSB; Laboratorios Conda, Madrid, Spain) containing 30% (*w/w*) glycerol (Sigma-Aldrich, Steinheim, Germany). *Clostridium* isolates were grown in Brain Heart Infusion Broth (BHIB, Biokar diagnostics) at 37 °C for 48 h under anaerobic conditions in a Whitley DG250 Anaerobic Workstation (Don Whitley Scientific, Bingley, England) and stored at −80 °C in BHIB containing 30% (*w/w*) glycerol. 

### 2.2. Preparation of Antimicrobial Solutions

Chitosan solution was prepared by dissolving 3% (*w/v*) chitosan (degree of deacetylation 90%, Aqua Premier Co., Chon Buri, Thailand) in 3% (*v/v*) acetic acid (Panreac, Barcelona, Spain), as described by Casquete et al. [24]. 

An ethanolic propolis extract (EPE) of 10 mg/mL was prepared using a sample of propolis collected in *Vila Franca, Viana do Castelo* (Portugal), and kept in the dark at −20 °C, according to Casquete et al. [25]. 

Solutions of nisin (25 μg/mL, Larbus, S.A., Madrid, Spain) were prepared in sterile distilled water.

### 2.3. Antimicrobial Activity and Minimum Inhibitory Concentration (MIC)

#### 2.3.1. Inoculum

Inocula of all microorganisms tested (Table 1) were prepared from a culture grown in TSA-YE at 37 °C, during 24 h under aerobic conditions, by suspending isolated colonies in sterile Ringer’s solution (Biokar diagnostics) to obtain turbidity equivalent to 0.5 in McFarland scale. *Clostridium* isolates were cultivated twice in BHIB at 37 °C for 48 h under anaerobic conditions.

#### 2.3.2. Determination of Antimicrobial Activity and MIC by Different Methods and pH Conditions

The antimicrobial activity of each compound was evaluated at concentrations described in Section 2.2. To determine the respective MIC, each compound was diluted in sterile distilled water, and the concentrations used varied between 0.0094% and 0.5% (*w/v*) for chitosan, 0.039 and 10 mg/mL for EPE, and 1.563 and 25 µg/mL for nisin. Minimum inhibitory concentrations were determined for all the compounds by the agar dilution and drop diffusion on agar methods. All the assays were performed in duplicate.

Additionally, culture media at pH values of 5, 6, and 7 were used to simulate different food products. The pH value of the culture medium was measured using a MicropH 2002 pHmeter (Crison, Barcelona, Spain) and adjusted to 5 and 6 with 0.1M HCl. 

##### Agar Dilution Method

Each compound and respective dilutions were incorporated in the TSA-YE. To each plate containing a given concentration, 10 µL of each inoculum was added (obtained as described in point Section 2.3.1). The same amounts of acetic acid and ethanol for each concentration of chitosan and propolis, respectively, were used as controls. Inocula grown in culture medium without incorporated compound were used as positive controls. The plates were incubated overnight at 37 °C, under aerobic conditions, except for *Clostridium* isolates, which were incubated for 48 h at 37 °C anaerobically. The lowest concentration of the compound that resulted in no visible growth on the surface of the culture medium was selected as the MIC of the compound.

##### Drop Diffusion on Agar Method

Each inoculum was evenly swabbed on a TSA-YE plate, and 10 µL of each compound and respective dilutions were dropped on the top of the plate. Acetic acid and ethanol were used as controls of chitosan and EPE solutions, and sterile distilled water was used as a negative control. The plates were incubated as described in *Agar dilution method* section, and the MIC of each compound was determined.

**Figure 1:**
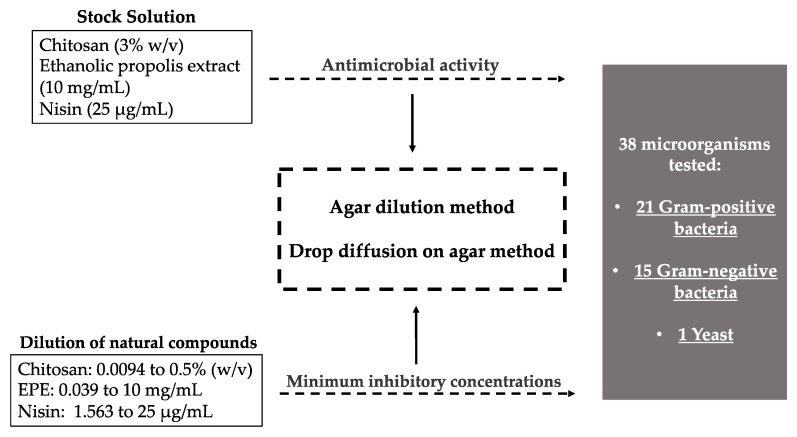
Schematic representation of the methodology used to determine antimicrobial activity and minimum inhibitory concentrations of three natural compounds.

The methodology described above is summarized in Figure 1.

### 2.4. Statistical Analysis 

All calculations were carried out using IBM SPSS Statistics software (version 27.0, IBM Corporation, Armonk, NY, USA). Significant differences between minimum inhibitory concentrations obtained by both techniques at each pH value were analyzed for each natural compound using the nonparametric Kruskal–Wallis test. Statistically significance was declared for *p* < 0.05.

## 3. Results and Discussion

### Antimicrobial Activity and Minimal Inhibitory Concentration

The antimicrobial activity of the various compounds was tested under different pH conditions to simulate their presence in different food products: (i) pH 5—fermented products, such as fermented sausages [26]; (ii) pH 6—ham or unfermented chorizo [27], and (iii) pH 7—products such as crab or shrimp that have a neutral pH [27] and also such as a control. It is important to highlight that for each pH condition, MIC was determined only for the microorganisms that are able to grow under these given conditions.

#### 3.1.1. Antimicrobial Activity and MIC of Chitosan

The stock solution of chitosan demonstrated antimicrobial activity against most of the microorganisms tested, by both the drop diffusion and agar dilution methods, under different pH conditions.

The results obtained for the minimum inhibitory concentrations of chitosan are shown in Table 2. It was possible to observe that chitosan inhibited Gram-positive and -negative bacteria and fungi. However, antimicrobial activity varied between different microorganisms. For several microorganisms, acetic acid had a greater inhibitory effect than chitosan at the same concentration. Still, for some microorganisms, under certain pH conditions, the MIC range of acetic acid coincided with the MIC of chitosan.

All the microorganisms were inhibited by chitosan in at least one of the conditions tested. For some Gram-positive and -negative microorganisms, at pH 5 and pH 6, chitosan did not have an inhibitory effect through the agar dilution method since acetic acid, individually, had a higher inhibitory effect. However, inhibition by the drop diffusion method was observed for the same microorganisms. The exceptions were all the Clostridium spp. isolates as well as Salmonella Braenderup ESB7 and Salmonella Enteritidis ESB8 since no inhibition was recorded by this method. This may be due to the fact that chitosan is likely to have less mobility in the agar mixture [28].

In general, considering the results in which the microorganisms were inhibited by both methods, except for those that were also inhibited by the acetic acid control, there was a greater inhibition by the agar dilution technique (*p* < 0.05) since lower concentrations of the compound were sufficient to inhibit the microorganisms, at all pH conditions. Klančnik et al. [29] also reported lower minimum inhibitory concentrations of plant extracts by the dilution method compared with the disc diffusion method. Unlike agar dilution, in both disc and drop diffusion methods, the size of the inhibition zone will be determined by how well the compound will be uniformly diffused in the agar medium, which can be problematic for many natural compounds insoluble in water [29].

In the study of Casquete et al. [24], the authors used the agar dilution method and obtained the MIC values of chitosan 0.02%, 0.2%, 0.02%, and 0.1% (*w/v*) for Staphylococcus aureus, Listeria innocua, Escherichia coli, and Salmonella Typhimurium, respectively. However, in this study, only the MIC value obtained for S. Typhimurium ESB9 was similar (0.15% *w/v*, at pH 7).

Jiang et al. [28] used the agar dilution method to determine the MIC values for two water-soluble chitosan derivatives against several microorganisms. The authors obtained the following results for chitosan derivatives with molecular weights of 43 and 67 kDa, respectively: 0.06% and 0.125% (*w/v*) for E. coli ATCC 25922; 0.25% and >1% (*w/v*) for S. Typhimurium; 0.25% and 0.25% (*w/v*) for Enterococcus faecalis ATCC 29212, and 0.125% and 0.125% (*w/v*) for Staph. aureus ATCC 29213. In the present study, similar MIC values were merely found at pH 7 for Staph. aureus ATCC 29213 and E. coli ATCC 25922, this last only for 67 kDa chitosan.

The results obtained support the knowledge that chitosan has antimicrobial activity against pathogenic and spoilage microorganisms, as already reported in other studies [2,30,31]. However, differences in antimicrobial activities may be due to several factors, including the type of chitosan, the degree of deacetylation, the molecular weight, and the bacterial strains. In addition, the pH value, strength, and presence of ionic solutes in the culture media may be capable of reacting with chitosan through molecular interactions and blocking the reactivity of the amine group and also the presence of substances that might interfere [24,32]. In this study, different pH values also influenced inhibition by chitosan, as well as the in vitro method used.

#### 3.1.2. Antimicrobial Activity and MIC of Ethanolic Propolis Extract (EPE)

Ethanolic propolis extract inhibited most of the microorganisms tested at a concentration of 10 mg/mL, by both the agar dilution and drop diffusion methods, under different pH conditions. At pH 5 and 6, all microorganisms were inhibited by the agar dilution method, while at pH 7, almost all microorganisms were inhibited. The concentration of 10 mg/mL at pH 5 inhibited Gram-positive microorganisms, yeast, and some Gram-negative microorganisms through the drop diffusion method. At pH 6 and 7, yeast, most Gram-positive microorganisms, and some Gram-negative microorganisms were inhibited.

In Table 3 are shown the minimum inhibitory concentrations of ethanolic propolis extract obtained. The 95% (*v/v*) ethanol was used as a control and showed no inhibition against most tested microorganisms under these conditions. This suggests that the inhibition of microorganisms is due to the compounds extracted from propolis. Under certain pH conditions, the MIC range of ethanol coincided with the MIC of propolis for some microorganisms in some values.

Significant differences (*p* < 0.05) were observed between minimum inhibitory concentrations obtained by both techniques at each studied pH. At pH 5, through the agar dilution method, all the microorganisms were inhibited by EPE. The acidic pH of the medium may contribute to the inhibitory effect. In the study of Lu et al. [33], the authors demonstrated that the antibacterial activity of EPE against *Staph. aureus* increased at pH 5. Under the same pH conditions, but using the drop diffusion agar method, most of the microorganisms tested in the present study were inhibited by EPE, and only the Gram-negative microorganisms *S.* Braenderup ESB7, *S*. Enteritidis ESB8, *S*. Typhimurium ESB9, *E. coli* ATCC 25922, *K. pneumoniae* ESB11, *P. vulgaris* ESB12, *P. mirabilis* ESB27, *Ps. aeruginosa* ESB13, and *Y. enterocolitica* ESB24 were not inhibited. Gram-negative microorganisms have an outer membrane and have a layer of lipopolysaccharides, which confers them greater resistance compared with Gram-positive microorganisms [34]. Results obtained at pH 6 were similar to those obtained at pH 5.

By the agar dilution method at pH 7, only the Gram-negative microorganisms *P. mirabilis* ESB27 and *Y. enterocolitica* NCTC 10406 were not inhibited. *Listeria innocua* 2030c was inhibited with 0.47 mg/mL of EEP, which is in agreement with the study of Silici et al. [35], in which the EPE used was inhibitory in concentrations between 0.25 and 0.50 mg/mL. However, the EPE used in the study by Casquete et al. [25] inhibited *L. innocua* at a concentration of 0.15 mg/mL.

Through the drop diffusion method at pH 7, EPE did not inhibit the Gram-negative microorganisms *A. baumannii* S-2 ESB32 and *A. calcoaceticus* S ESB31 and Gram-positive *G. stearothermophilus* ESB16, *Staph. aureus* 18N, and *Clostridium* spp., as well as the same microorganisms that were not inhibited at pH 5 by the same method. *Bacillus cereus* ESB14 isolate was inhibited by EPE at 0.47–2.5 mg/mL. Kim and Chung [36] demonstrated that *B. cereus* was inhibited by Korean propolis with a concentration of 0.036 mg/mL, much lower than that observed in this study. The same occurred for *Staph. aureus* strains (MIC 0.072 mg/mL) and a *L. monocytogenes* strain (MIC 0.14 mg/mL). However, it should be noted that the origin of propolis was different. As in the study of Kim and Chung [36], Gram-negative bacteria *S*. Typhimurium ESB9 and *E. coli* ATCC 25922 were not inhibited by EPE. Akca et al. [37] reported that, in the planktonic state, *Ent. faecalis* ATCC 29212 was inhibited by propolis concentration of 0.008 mg/mL, by agar dilution method, much lower than obtained in this study (0.94 mg/mL, at pH 7). On the other hand, Pamplona-Zomenhan et al. [38] demonstrated that *Staph. aureus* ATCC (MSSA—29213 and MRSA—33511) was inhibited by an ethanolic propolis extract from Brazil at a concentration of 1.42 mg/mL, by the agar dilution method.

Overall, it was possible to observe that ethanolic propolis extract can inhibit Gram-positive and -negative bacteria and yeast. Akca et al. [37] reported that propolis was more effective in inhibiting Gram-positive than Gram-negative bacteria, which is in agreement with this study. It was also possible to notice that the results obtained by the agar dilution method were quite different from those of the drop diffusion method. Unlike the drop diffusion method, almost all Gram-negative bacteria were inhibited by EPE through the agar dilution method. For most microorganisms, the MIC values were lower in the agar dilution method. The antimicrobial and antifungal activity of propolis is influenced by the product’s origin, chemical composition, dose, extraction, and solvent preparation [39], and the probable presence of nonvolatile components of the extract [36]. Besides the antimicrobial activity of propolis, the advantage of the presence of many of its constituents in food and their recognition as safe substances makes it a potential food preservative [9,40].

#### 3.1.3. Antimicrobial Activity and MIC of Nisin

Unlike Gram-negative bacteria, most Gram-positive bacteria were inhibited by nisin at a concentration of 25 µg/mL, both by the agar dilution and by the drop diffusion method, under different pH conditions.

Table 4 shows the minimum inhibitory concentrations of nisin against all the target microorganisms tested. Significant differences (*p* < 0.05) were observed between minimum inhibitory concentrations obtained by both techniques at pH 5 and 7. At pH 6, no significant differences (*p* = 0.052) were found between both techniques used.

It is clear that nisin inhibited a greater number of microorganisms at a more acidic pH using the agar dilution method (Table 4). The antimicrobial activity of nisin is strongly influenced by the pH of the solution [20], with its most significant activity observed at acidic pH (2 to 3), followed by the drastic loss of activity at higher pH values [41]. As in the agar dilution method, the compound was incorporated in the medium, and the direct contact with an acid solution may have allowed a greater inhibition of a more significant number of microorganisms at pH 5 than at pH 6 and 7. Using this technique, four strains of *Acinetobacter* were also inhibited at pH 5. Zheng et al. [42] also demonstrated the inhibition of an *A. calcoaceticus* strain by nisin at 37 ºC, when using the disk diffusion in agar. The same did not happen in the drop diffusion method since only Gram-positive microorganisms were inhibited at different pH values.

According to the literature, nisin only inhibits Gram-positive bacteria since Gram-negative bacteria have a natural resistance to this bacteriocin [34,43,44]. Gram-positive spore-forming microorganisms such as *Bacillus* and *Clostridium* are particularly susceptible to nisin, with spores being more sensitive than vegetative cells [45,46]. In addition to these microorganisms, nisin acts against *L. monocytogenes* and lactic acid bacteria [45,47]. On the other hand, yeasts are resistant to nisin, and, for this reason, nisin can be applied together with yeasts in fermentation to control the growth of lactic acid bacteria [45].

The inhibition of some Gram-negative bacteria may have been a consequence of sensitizing the strains of *Acinetobacter* to the acidic environment of the culture medium since the temperature and pH values may alter the bacteriocin activity and/or the permeability of the outer membrane [34]. The exposure of Gram-negative bacteria, such as *E. coli* or *S.* Typhimurium, to conditions of low temperature and low pH favors the production of a cell envelope that is more susceptible to the action of bacteriocins, allowing the action of nisin under conditions of prolonged exposure [48,49]. However, to inhibit more Gram-negative bacteria, nisin could be combined with chelating agents, such as ethylenediamine tetra-acetic acid (EDTA) [34,50], despite being a strategy not yet used commercially. The chelating agent acts by destabilizing the outer membrane, in part by releasing the lipopolysaccharides layer, allowing bacteriocin to access the plasma membrane [51]. In general, a low concentration of EDTA is necessary to sensitize Gram-negative microorganisms to bacteriocins. In the study by Prudêncio et al. [34], it was demonstrated that *S*. Typhimurium became susceptible to nisin with 1.5 mM EDTA. However, for the application in food, possibly more significant amounts of EDTA and bacteriocin will be necessary due to the chelator’s binding to exogenous divalent cations and nonspecific interactions of nisin with food components [34]. Additionally, nisin and chitosan [52,53] or nisin and essential oils [54,55] could be combined to increase the sensitivity of bacteria. Nisin could also be incorporated into coatings, packaging films [9], and nisin-loaded nanoparticles [56].

The study of antimicrobial activity of natural compounds has several limitations, particularly related to the techniques used for its determination. The present study is not an exception, and it intends to reinforce those limitations and the need for standardized techniques. In the drop diffusion on agar method, it is supposed that compound diffuses freely and uniformly in the solid agar medium, but when the natural compound has less solubility in water, it could diffuse more slowly into the culture medium and can give rise to inaccurate conclusions [22,29]. Beyond that, the diffusion methods could be laborious and time-intensive, like all agar-based methods [29]. In the agar dilution method, the mobility of the natural compound in the agar mixture could also be problematic [28]. There are standardized methods by the CLSI or the EUCAST, although the published guides refer to antimicrobials of clinical use. Moreover, different growth conditions to simulate different food matrices are not included in those guides, and natural antimicrobials could behave differently in different matrices. Therefore, it is crucial to define the standard in vitro techniques for determining minimum inhibitory concentrations of natural antimicrobial compounds before their in situ application in food matrices.

## 4. Conclusions

In the present study, it was possible to support the knowledge that the natural compounds propolis, chitosan, and nisin have antimicrobial activity against various pathogenic and spoilage microorganisms. With the natural compounds used, a greater number of Gram-positive microorganisms was inhibited compared with Gram-negative microorganisms. Most of the microorganisms were inhibited by chitosan and propolis, and, in contrast, most of the Gram-positive bacteria, by nisin. It was also possible to conclude that different pH values of the testing media and different in vitro determination methods influenced the antimicrobial activity of the compounds. Lower minimum inhibitory concentrations were obtained at lower pH values and for the agar dilution method. Beyond that, some microorganisms inhibited by the natural compounds on the agar dilution method were not inhibited by the same compounds and at the same concentrations on the drop diffusion technique. 

Since natural compounds are increasingly in demand as a promising alternative to chemical preservatives in food preservation, it is crucial to define an in vitro standard method to assess their antimicrobial activity in order to compare different samples and studies.

## Figures and Tables

**Table 1:** Microorganisms used in this study.

Microorganisms	Strains	Source
Gram-positive	- *Bacillus cereus* ESB14 - *Bacillus subtilis* ESB15 - *Geobacillus* *stearothermophilus* ESB16 -*Staphylococcus aureus* 18N (Methicillin-resistant *Staphylococcus aureus*—MRSA) - *Staphylococcus aureus* 2037M1 (MSSA) - *Clostridium sporogenes* 1.31 - *Clostridium sporogenes* 1.34 - *Clostridium sporogenes* 1.61 - *Clostridium perfringens* 1.16 - *Clostridium perfringens* 1.19 - *Clostridium perfringens* 1.22 - *Listeria monocytogenes* 7946 - *Listeria monocytogenes* 7947	Isolates from Culture Collection of *Escola Superior de Biotecnologia* (Porto, Portugal)
	- *Listeria innocua* 2030c	*Public Health Laboratory Service* (PHLS; London, UK)
	- *Enterococcus faecalis* ATCC 29212 - *Staphylococcus aureus* ATCC 29213	*American Type Culture Collection* (ATCC; Manassas, VA, USA)
	- *Enterococcus faecalis* DSMZ 12956 - *Enterococcus faecium* DSMZ 13590 *- Enterococcus flavescens* DSMZ 7370 - *Enterococcus gallinarum* DSMZ 20628 *- Enterococcus casseliflavus* DSMZ 20680	*Leibniz Institute DSMZ—German Collection of Microorganisms and Cell Cultures* (DSMZ; Braunschweig, Germany)
Gram-negative	- *Salmonella* Braenderup ESB7 - *Salmonella* Enteritidis ESB8 - *Salmonella* Typhimurium ESB9 - *Klebsiella pneumoniae* ESB11 - *Proteus vulgaris* ESB12 - *Proteus mirabilis* ESB27 - *Pseudomonas aeruginosa* ESB13 - *Yersinia enterocolitica* ESB24 - *Acinetobacter baumannii* R ESB28 - *Acinetobacter baumannii* S—1 ESB29 - *Acinetobacter baumannii* S—2 ESB32 - *Acinetobacter calcoaceticus* R ESB30 - *Acinetobacter calcoaceticus* S ESB31	Isolates from Culture Collection of *Escola Superior de Biotecnologia* (Porto, Portugal)
	- *Escherichia coli* ATCC 25922	*American Type Culture Collection* (ATCC; Manassas, VA, USA)
	- *Yersinia enterocolitica* NCTC 10406	*National Collection of Type Cultures—Culture Collection of Public Health England* (NCTC; Salisbury, UK)
Fungi	- *Saccharomyces cerevisiae* ESB26	Isolates from Culture Collection of *Escola Superior de Biotecnologia* (Porto, Portugal)

S—Microorganisms sensitive to antibiotics; R—Microorganisms resistant to antibiotics; Note: Isolates from Culture Collection of *Escola Superior de Biotecnologia* can be made available on request.

**Table 2:** Minimum inhibitory concentration (MIC) of chitosan solution (% *w/v*) against various microorganisms, obtained through the agar dilution and drop diffusion methods at pH values of 5, 6 and 7.

Microorganisms	MIC (% *w/v*)					
	pH 5		pH 6		pH 7	
	Agar Dilution	Drop Diffusion	Agar Dilution	Drop Diffusion	Agar Dilution	Drop Diffusion
**Gram-positive bacteria**						
*Ent. faecalis* ATCC 29212	^(1)^	0.3–0.35	0.15	0.15	0.1	0.075
*Ent. faecalis* DSMZ 12956	0.035–0.15 ^(2)^	0.075	0.08	0.075–0.15	0.04–0.065	0.15–0.3
*Ent. faecium* DSMZ 13590	0.06–0.15 ^(2)^	0.075–0.15	0.08	0.15	0.07	0.15
*Ent. flavescens* DSMZ 7370	0.09–0.15 ^(2)^	0.075–0.15	0.15 ^(2)^	0.15	0.08–0.085 ^(2)^	0.075–0.15
*Ent. gallinarum* DSMZ 20628	^(1)^	0.0375–0.075	^(1)^	0.15	0.15 ^(2)^	0.15
*Ent. casseliflavus* DSMZ 20680	^(1)^	0.3 ^(2)^	^(1)^	0.15–0.3	0.07 ^(2)^	0.075
*B. cereus* ESB14	^(1)^	0.075 ^(2)^	^(1)^	0.15	0.07 ^(2)^	0.15
*B. subtilis* ESB54	^(1)^	0.15–0.3	^(1)^	0.3	0.15 ^(2)^	^(1)^
*G. stearothermophilus* ESB16	^(1)^	0.075	^(1)^	*	0.065 ^(2)^	*
*Staph. aureus* ATCC 29213	^(1)^	0.3	^(1)^	0.3	0.15–0.3 ^(2)^	0.3
*Staph. aureus* 18N (MRSA)	^(1)^	*	^(1)^	*	0.15	*
*Staph. aureus* 2037 M1(MSSA)	^(1)^	*	^(1)^	*	0.15 ^(2)^	0.3
*L. monocytogenes* 7946	^(1)^	0.0375–0.075	^(1)^	0.15–0.3	0.08–0.09 ^(2)^	^(1)^
*L. monocytogenes* 7947	^(1)^	0.0375–0.075 ^(2)^	^(1)^	0.075–0.15	0.08–0.085	0.15
*L. innocua* 2030c	^(1)^	0.075	^(1)^	0.15	0.08–0.1 ^(2)^	0.15
*Cl. sporogenes* 1.31	0.075 ^(2)^	*	0.0375 ^(2)^	*	0.15 ^(2)^	*
*Cl. sporogenes* 1.34	0.075 ^(2)^	*	0.0375 ^(2)^	*	0.15 ^(2)^	*
*Cl. sporogenes* 1.61	0.075 ^(2)^	*	0.0375 ^(2)^	*	0.15 ^(2)^	*
*Cl. perfringens* 1.16	0.075	*	0.0375	*	0.15 ^(2)^	*
*Cl. perfringens* 1.19	0.075 ^(2)^	*	0.0375	*	0.15 ^(2)^	*
*Cl. perfringens* 1.22	0.075 ^(2)^	*	0.0375	*	0.15 ^(2)^	*
**Gram-negative bacteria**						
*Salmonella* Braenderup ESB7	^(1)^	0.15–0.3^(2)^	^(1)^	0.3–0.35^(^^2)^	^(1)^	0.3–0.45^(2)^
*S*. Enteritidis ESB8	^(1)^	0.15^(2)^	^(1)^	0.15	^(1)^	0.3–0.5^(2)^
*S.* Typhimurium ESB9	^(1)^	0.15	^(1)^	0.15	0.15 ^(2)^	^(1)^
*E. coli* ATCC 25922	^(1)^	0.15–0.3 ^(2)^	^(1)^	0.3–0.35	0.15 ^(2)^	^(1)^
*Klebsiella pneumoniae* ESB11	^(1)^	0.15^(2)^	^(1)^	0.15	^(1)^	0.3 ^(2)^
*Proteus vulgaris* ESB12	^(1)^	0.15^(2)^	^(1)^	0.15–0.3 ^(2)^	^(1)^	0.3–0.35^(2)^
*P. mirabilis* ESB27	^(1)^	0.15 ^(2)^	0.1 ^(2)^	^(1)^	0.055 ^(2)^	0.15
*Ps. aeruginosa* ESB13	^(1)^	0.15 ^(2)^	^(1)^	0.3 ^(2)^	0.1–0.3 ^(2)^	^(1)^
*Yersinia enterocolotica* NCTC 10406	^(1)^	0.15–0.3 ^(2)^	^(1)^	0.3–0.35^(2)^	^(1)^	0.3–0.35^(2)^
*Y. enterocolitica* ESB24	0.075–0.09 ^(2)^	0.15 ^(2)^	^(1)^	0.3–0.35 ^(2)^	0.075	0.3 ^(2)^
*A. baumannii* R ESB28	^(1)^	0.15 ^(2)^	^(1)^	0.15–0.3 ^(2)^	0.08–0.1 ^(2)^	0.15–0.3
*A. baumannii* S–1 ESB29	^(1)^	0.15	^(1)^	0.075–0.15 ^(2)^	0.08	0.075
*A. baumannii* S–2 ESB32	^(1)^	*	^(1)^	0.075–0.15	0.08–0.1 ^(2)^	0.15–0.3
*A. calcoaceticus* R ESB30	^(1)^	0.3 ^(2)^	^(1)^	0.15–0.3 ^(2)^	0.08–0.1 ^(2)^	0.15–0.3
*A. calcoaceticus* S ESB31	^(1)^	^(1)^	^(1)^	0.0375–0.15 ^(2)^	0.08–0.095 ^(2)^	0.15 ^(2)^
**Yeast**						
*Sac. cerevisiae* ESB26	0.15	*	0.15	0.35	0.02–0.08	0.3–0.5 ^(2)^

^(1)^ Acetic acid individually demonstrated a more inhibitory effect than the chitosan solution; ^(2)^ MIC range of acetic acid coincides with that of chitosan solution, at specific values; * no inhibition.

**Table 3:** Minimum inhibitory concentration (MIC) of ethanolic propolis extract (mg/mL) against various microorganisms, obtained through the agar dilution and drop diffusion methods at pH values of 5, 6 and 7.

Microorganisms	MIC (mg/mL)					
	pH5		pH6		pH7	
	Agar Dilution	Drop Diffusion	Agar Dilution	Drop Diffusion	Agar Dilution	Drop Diffusion
**Gram-positive bacteria**						
*Ent. faecalis* ATCC 29212	0.078	1.25–2.5	0.156–0.625	2	0.94	0.47
*Ent. faecalis* DSMZ 12956	0.078	2.5	0.156–0.625	2.5–3.5	0.63	1.25–5
*Ent. faecium* DSMZ 13590	0.078	1.25–2.5	0.156–0.625	2.5	0.47–1.25	1.25–2.5
*Ent. flavescens* DSMZ 7370	0.078	0.94–2.5	0.233–0.625	1.25	0.94	5
*Ent. gallinarum* DSMZ 20628	0.31	0.625–0.94	0.233–0.625	1.25–2	1.25–1.875	2
*Ent. casseliflavus* DSMZ 20680	0.078	0.625–0.94	0.233–0.625	0.94–2.5	1.25	1.875
*B. cereus* ESB14	<0.039	0.156	0.059	0.625	0.156–0.625	0.47–2.5
*B. subtilis* ESB54	0.078	1.25–1.875	0.233–0.625	1.25–2.5	1.25	1.25
*G. stearothermophilus* ESB16	<0.039 ^(1)^	5	<0.039	5–10	0.156–0.625	*
*Staph. aureus* ATCC 29213	0.078	1.25	0.078	1.25–2.5	0.47	5
*Staph. aureus* 18N (MRSA)	0.078	2.5	0.078	2.5–5	0.31–0.625	*
*Staph. aureus* 2037 M1(MSSA)	0.117	1.25–2.5	0.078	1.25–2.5	0.31–0.625	2.5
*L. monocytogenes* 7946	0.117	0.31	0.117	1.25	0.47	2.5–5
*L. monocytogenes* 7947	0.078	0.625–0.94	0.117	0.94–2.5	0.47	1.25–1.875
*L. innocua* 2030c	0.156	0.625–1.25	0.117–0.625	1.875	0.47	2.5–3.5
*Cl. sporogenes* 1.31	0.31	0.31	0.078	0.625	0.31	*
*Cl. sporogenes* 1.34	0.31	0.31	0.078	0.625	0.31	*
*Cl. sporogenes* 1.61	0.31	0.31	0.078	0.625	0.31	*
*Cl. perfringens* 1.16	0.31	5	0.156	1.25	0.31	*
*Cl. perfringens* 1.19	0.31	10	0.156	*	0.31	*
*Cl. perfringens* 1.22	0.31	5	0.156	0.625	0.31	*
**Gram-negative bacteria**						
*Salmonella* Braenderup ESB7	4.5–10 ^(1)^	*	3–10	*	10	*
*S*. Enteritidis ESB8	4.5–10 ^(1)^	*	3–10	*	10	*
*S.* Typhimurium ESB9	3.5	*	2–10 ^(1)^	*	10	*
*E. coli* ATCC 25922	4.5	*	4–10	*	10	*
*Klebsiella pneumoniae* ESB11	5–10 ^(1)^	*	3–10	*	10	*
*Proteus vulgaris* ESB12	4.5–10 ^(1)^	*	3–10	*	10	*
*P. mirabilis* ESB27	5–5.5	*	5.5	*	*	*
*Ps. aeruginosa* ESB13	4.5	*	3–10	*	6.5	*
*Yersinia enterocolotica NCTC 10406*	10	*	5	*	*	*
*Y. enterocolitica* ESB24	0.078–1.25	*	0.233–5	*	0.47–5	*
*A. baumannii* R ESB28	2–5	5–10	2.5–5	*	5–10 ^(1)^	10
*A. baumannii* S–1 ESB29	0.63	3	2–5	10	4.5–10	10
*A. baumannii* S–2 ESB32	2–5	5–10	2.5–5	3	5.5	*
*A. calcoaceticus* R ESB30	0.47	0.63–5	2–5	4	4.5–10 ^(1)^	10
*A. calcoaceticus* S ESB31	0.31–1.25	5–10	2–5	5–10	4.5–10 ^(1)^	*
**Yeast**						
*Sac. cerevisiae* ESB26	0.117	0.625–1.25	0.117	0.47	0.31–0.625	0.625–1.25

^(1)^ The range of the minimum inhibitory concentration of ethanol coincides with that of propolis, in specific values; * no inhibition.

**Table 4:** Minimum inhibitory concentration (MIC) of nisin (µg/mL) against various microorganisms, obtained through the agar dilution and drop diffusion methods at pH values of 5, 6 and 7.

Microorganisms	MIC (µg/mL)					
	pH5		pH6		pH7	
	Agar Dilution	Drop Diffusion	Agar Dilution	Drop Diffusion	Agar Dilution	Drop Diffusion
**Gram-positive bacteria**						
*Ent. faecalis* ATCC 29212	*	*	*	12.5	*	5
*Ent. faecalis* DSMZ 12956	12.5	6.25–12.5	15	12.5	12.5–25	5
*Ent. faecium* DSMZ 13590	12.5	6.25–12.5	25	6.25–12.5	12.5–25	5
*Ent. flavescens* DSMZ 7370	12.5	3.125–6.25	5–25	12.5	25	8
*Ent. gallinarum* DSMZ 20628	3.125	6.25–12.5	3.125	10	5	12.5
*Ent. casseliflavus* DSMZ 20680	<1.563	6.25	6.25–12.5	8	6.25	12.5
*B. cereus* ESB14	5–25	25	*	*	*	12.5–25
*B. subtilis* ESB54	12.5–25	25	*	10	*	8
*G. stearothermophilus* ESB16	5–25	25	*	*	*	*
*Staph. aureus* ATCC 29213	*	*	*	*	*	*
*Staph. aureus* 18N (MRSA)	*	*	*	*	*	25
*Staph. aureus* 2037 M1(MSSA)	15	*	20	25	8–25	20
*L. monocytogenes* 7946	6.25	12.5	12.5–15	8	*	10–25
*L. monocytogenes* 7947	6.25	6.25–12.5	15	5	20	10
*L. innocua* 2030c	5–25	10	*	5	*	12.5–25
*Cl. sporogenes* 1.31	6.25	25	*	*	*	*
*Cl. sporogenes* 1.34	25	25	*	*	*	*
*Cl. sporogenes* 1.61	25	*	*	*	*	*
*Cl. perfringens* 1.16	6.25	*	12.5	*	25	*
*Cl. perfringens* 1.19	25	*	*	6.25	*	*
*Cl. perfringens* 1.22	25	*	25	*	*	*
**Gram-negative bacteria**						
*Salmonella* Braenderup ESB7	*	*	*	*	*	*
*S*. Enteritidis ESB8	*	*	*	*	*	*
*S.* Typhimurium ESB9	*	*	*	*	*	*
*E. coli* ATCC 25922	*	*	*	*	*	*
*Klebsiella pneumoniae* ESB11	*	*	*	*	*	*
*Proteus vulgaris* ESB12	*	*	*	*	*	*
*P. mirabilis* ESB27	*	*	*	*	*	*
*Ps. aeruginosa* ESB13	*	*	*	*	*	*
*Yersinia enterocolotica* NCTC 10406	*	*	*	*	*	*
*Y. enterocolitica* ESB24	*	*	*	*	*	*
*A. baumannii* R ESB28	12.5–25	*	*	*	*	*
*A. baumannii* S–1 ESB29	8–25	*	*	*	*	*
*A. baumannii* S–2 ESB32	*	*	*	*	*	*
*A. calcoaceticus* R ESB30	12.5–25	*	*	*	*	*
*A. calcoaceticus* S ESB31	5–25	*	*	*	*	*
**Yeast**						
*Sac. cerevisiae* ESB26	*	*	*	*	*	*

* No inhibition.
